# Management of full-length complete ureteral avulsion

**DOI:** 10.1590/S1677-5538.IBJU.2015.0372

**Published:** 2016

**Authors:** Kaifa Tang, Fa Sun, Yuan Tian, Yili Zhao

**Affiliations:** 1 Department of Urology, Affiliated Hospital of Guizhou Medical University, Guiyang, China

**Keywords:** Ureteral avulsion, Greater omentum, Pyeloureterostomy, Ureterovesical anastomosis

## Abstract

**Introduction:**

Complete ureteral avulsion is one of the most serious complications of ureteroscopy. The aim of this report was to look for a good solution to full-length complete ureteral avulsion.

**Case presentation:**

A 40-year-old man underwent ureteroscopic management. Full-length complete avulsion of ureter occurred during ureteroscopy. Pyeloureterostomy plus greater omentum investment outside the avulsed ureter and ureterovesical anastomosis were performed 6 hours after ureteral avulsion. The patient was followed-up during 34 months. Double-J tube was removed at 3 months after operation. Twenty three months after the first operation, the patient developed hydronephrosis because of a new ureter upside stone, then rigid ureteroscopy and holmium laser lithotripsy were used successfully.

**Conclusion:**

Pyeloureterostomy plus greater omentum investment outside the avulsed ureter and ureterovesical anastomosis may be a good choice for full-length complete ureteral avulsion.

## INTRODUCTION

Urolithiasis is one of the most common diseases of urinary system. With the wide application of ureteroscopes, percutaneous nephroscopes, and endoscopic stone extractors, the incidence of iatrogenic ureteral avulsion tends to grow year by year (1). Ureteral avulsion refers to discontinuation of the full thickness of the ureter. Inappropriate management of this serious condition may lead to nephrectomy (2). How to manage ureteral avulsion has become a challenge to urologists. Here, we presented the management of full-length complete ureteral avulsion.

### Case presentation

**Figure 1:**
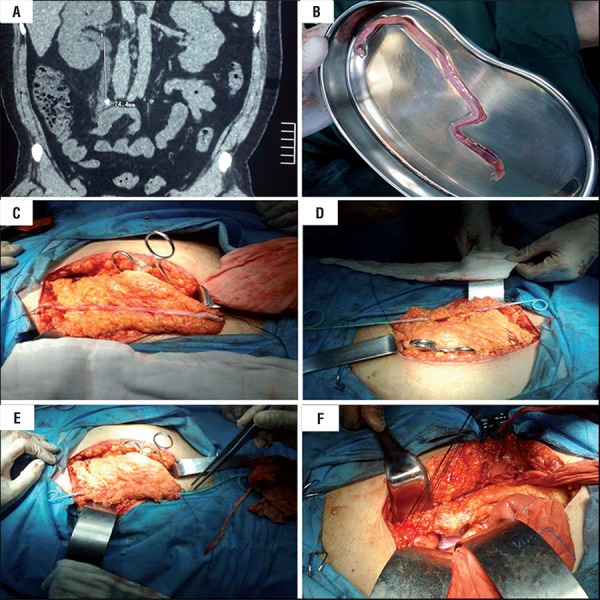
A) Right hydronephrosis secondary to a stone located in the ureter; B) The avulsed ureter; C) Free vascularized greater omentum in order to adapt to the avulsed ureter length; D) A single double-J stent tube was placed inside the ureter, package of ureter with greater omentum from the inside to the outside; E) The greater omentum was sutured around the ureter closely; F) pyeloureterostomy and ureterovesical anastomosis, respectively.

A 40-year-old male presented to us with right flank pain experienced for two weeks. Pain was colicky in nature, radiating to genitalia, associated with vomiting. Bowel habits were normal. There was no history suggestive of any other system involvement. Examination was unremarkable. Computed Tomography (CT) of urinary system revealed right hydronephrosis and a calculus measured 0.9x0.8x0.6cm located in the right upper ureter, and the distance between the stone and renal pelvis was 7.44cm (Figure-1a).

Ureteroscopic removal was planned. Forceful placement of rigid ureteroscope resulted in instrument drag, which hampered its maneuverability. An attempt at extraction produced full-length complete avulsion of ureter. The avulsed ureter was pulled out of body (Figure-1b), and the native ureter was preserved in physiological saline. The reconstruction treatment selection was a decision made for the patient after extensive discussion with urologists of the Affiliated Hospital of Guizhou Medical University. After discussing the complication with the patient, his spouse and his family members, we underwent ureteral reconstruction by standard open surgical techniques. About 6 hours after ureteral avulsion, pyeloureterostomy plus greater omentum investment outside the avulsed ureter and ureterovesical anastomosis were performed for the patient. A single double-J stent tube (6F, Budd Company) was placed inside the ureter (Figures 1c-f).

**Figures 2:**
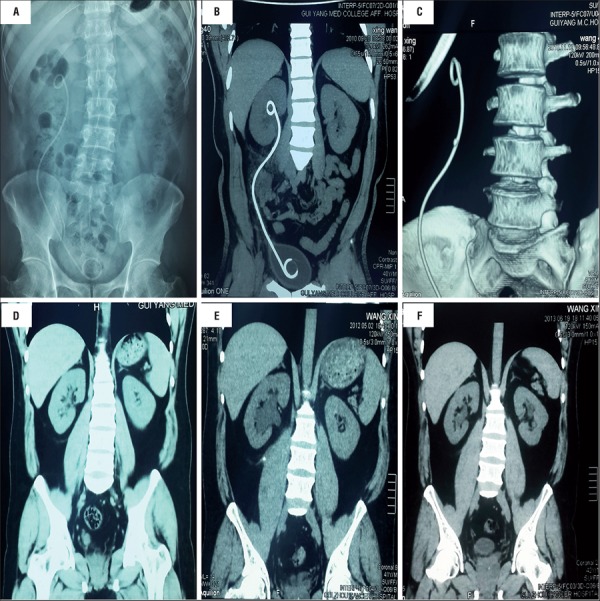
A and B) KUB and CT at one month; C) CT indicated that there was a stone like-material attached to the double-J tube at 3 months; D) CT indicated that there were no right hydronephrosis or other abnormalities at 5 months; E) CT revealed hydronephrosis secondary to a stone located in the upper ureter at 23 months; F) CT of urinary system revealed no hydronephrosis, renal atrophy or other complications at 34 months.

The patient was followed-up for 34 months. Plain abdominal radiography (KUB) and CT indicated that there was no hydronephrosis and the position of double-J tube was normal (Figures 2a and b). At 3 months, CT indicated that there was a stone like-material attached to the double-J tube (Figure-2c). After extensive discussion with urologists and with the patient, his spouse and his family members, we decided to pull out the double-J tube finally. At 5 months, CT indicated that there were no hydronephrosis and other abnormalities (Figure-2d). At 23 months after first operation, CT revealed right hydronephrosis and a new upper ureteral stone (Figure-2e). Rigid ureteroscopy and holmium laser lithotripsy were used, and a single double-J stent tube was placed inside the ureter after management, which was removed one month later. At 34 months, CT of urinary system revealed no hydronephrosis, renal atrophy or other complication (Figure-2f).

## DISCUSSION

Urolithiasis is a very common and major disease in urology department, the lifetime risk of urolithiasis in the general population is 13% (3, 4). Ureteroscopy is considered a reasonable therapeutic option for patients with ureteral stones (5). However, ureteroscopic examination or treatment procedures may lead to various complications, such as stone residuals, mucosa injury, perforation, bleeding, and edema (6). Ureteral avulsion is a rare but extremely serious complication, incidence of which has been reported at 0-3.75% (7), which is very difficult to manage. Many treatments may be considered: autotransplantation of kidney, ureterovesical anastomosis; replacement of the ureter with the ileum, ureterocalicostomy; and ureteral-ureteral end-end anastomosis, extended spiral bladder flap treatment of upper ureteral loss, pyeloureterostomy plus greater omentum investment outside the avulsed ureter and ureterovesical anastomosis and so on (8-11). The pros and cons of all treatment options in the management of ureteral avulsion are listed in Table-1. The actual surgical procedure depends on the site and severity of injury.

**Table 1:** The pros and cons of all treatment options in the management of ureteral avulsion.

Methods of reconstruction	Pros	Cons
Autotransplantation of kidney (12)	Priority selection for isolated kidney, renal insufficiency and complete ureteral avulsion	The operation was difficulty, and patients and their families is difficult to accept
Ureterovesical anastomosis, ureterocalicostomy and Ureteral-ureteral end-end anastomosis (9, 13)	The operation was simple, less trauma	Anastomosis stenosis or leakage, and not suitable for complete ureteral avulsion
Replacement of the ureter with the ileum (14, 15)	High success rate	Obstruction, delayed formation of mucus, stones, recurrent infection, ischemic necrosis of intestine, electrolyte disorder and preoperative bowel preparation
Extended spiral bladder flap treatment of upper ureteral loss (11)	The recovery of renal function was good, less complications	Repair ureter injury length is limited
Pyeloureterostomy plus greater omentum investment outside the avulsed ureter and ureterovesical anastomosis (8)	The renal function recovered well, especially suitable for full-length ureteral avulsion	The operation was difficulty, fibrosis tissue was forming outside ureter and anastomotic atresia of ureter-bladder
Nephrectomy (7)	The operation was easily	Patients and their families are difficult to accept

The treatment of ureteral avulsion is challenging and remains controversial. According to this case report with full-length complete ureteral avulsion, it is suitable for pyeloureterostomy plus greater omentum investment outside the avulsed ureter and ureterovesical anastomosis. Previous studies showed that the blood supplies of greater omentum could nourish the avulsed ureter (8, 9).

We believe that pyeloureterostomy plus greater omentum investment outside the avulsed ureter and ureterovesical anastomosis may be a good solution to full-length complete ureteral avulsion.

## CONSENT

Written informed consent was obtained from the patient for publication of this case report and any accompanying images. A copy of the written consent is available for review by the Editor of this journal.
